# Effect of Mare Nectaris on Feng-flavor Baijiu: an insight into the sensory evaluation and flavor analysis

**DOI:** 10.3389/fnut.2025.1684573

**Published:** 2025-12-05

**Authors:** Yuqi Chen, Jinghui Hu, Feng Zhou, Xinli Meng, Xuesong Hu, Jiaojiao Zhang, Xinglin Han

**Affiliations:** 1 Liquor-Making Engineering Research and Development Department, China National Research Institute of Food and Fermentation Industries Co., Ltd., Beijing, China; 2 Liu Lin Liquor Group Co., Ltd., Baoji, Shanxi, China

**Keywords:** Feng-flavor Baijiu, Mare Nectaris, storage time, sensory evaluation, flavor analysis

## Abstract

Feng-flavor Baijiu is a significant subtype of traditional Chinese baijiu that has considerable consumer popularity. This study examined how the quality of Feng-flavor Baijiu was affected by the various times that Mare Nectaris was used. The findings indicated that baijiu kept for a year in older Mare Nectaris (>10 years) was better than that in younger ones (< 5 years). Older Baijiu had richer honey, fruity, and Mare Nectaris-aromas, whereas fresh Baijiu had an astringent, lime flavor. Later in storage, old Mare Nectaris had a stronger effect on volatile taste constituents. OAVs >1 were present in 30 compounds in the old Mare Nectaris and 27 in the new ones. Propanal, lactic acid, acetaldehyde, and active amyl alcohol are linked to the “honey aroma.” This study may provide a theoretical basis for selecting suitable Mare Nectaris to enhance Feng-flavor Baijiu's flavor and sensory appeal.

## Graphical Abstract

**Figure:**
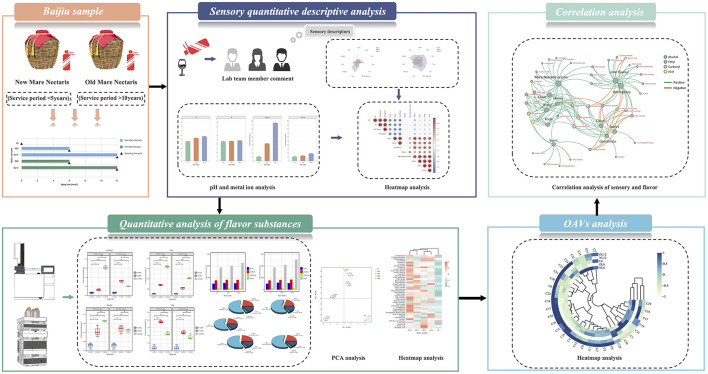

## 1 Introduction

Chinese traditional fermented Baijiu is a renowned alcoholic beverage with a unique flavor. Its distinctive charm lies in the complex interaction of trace components that form a unique aroma and taste (1). The Feng-flavor Baijiu is one of the twelve main fragrance types. Feng-flavor Baijiu is prized for its mellow flavor, sweetness, and lingering aftertaste, as well as its combination scent of ethyl acetate and ethyl hexanoate (2, 3). The process of aging is essential for distilled spirits because it gives Baijiu its distinct flavors, colors, and scents (4). Traditional storage containers for Baijiu mainly include earthenware jars and “Mare Nectaris.” The use of Mare Nectaris for storing baijiu in China has a history of nearly a 1,000 years, starting in the Tang Dynasty and flourishing in the Song Dynasty. In the brewing process of Feng-flavor Baijiu, storage in Mare Nectaris is a key step in shaping its unique flavor. A Mare Nectaris is a large traditional storage container made through complex techniques.

**Figure 1:**
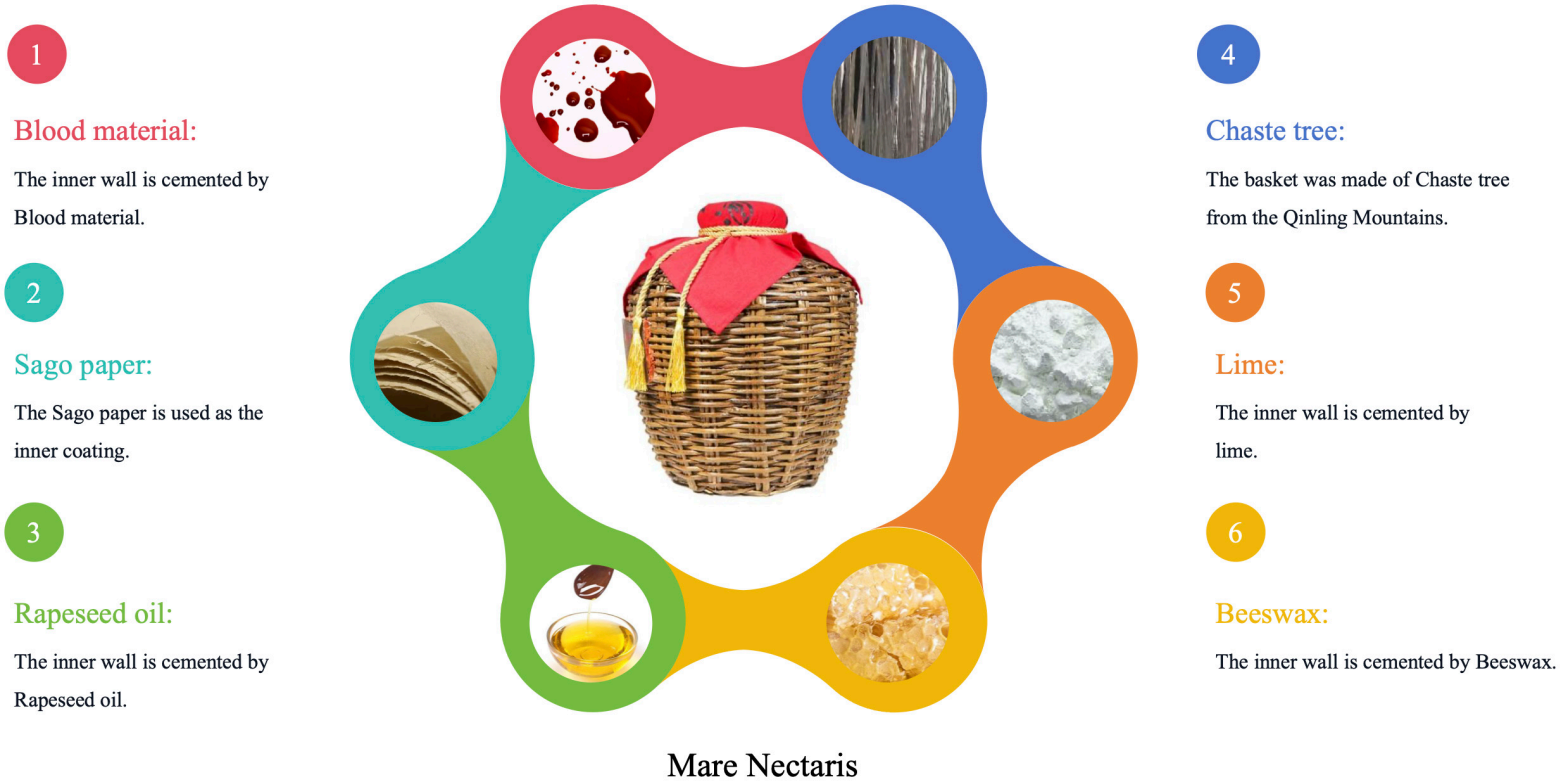
The materials for making Mare Nectaris.

Mare Nectaris are made of Jingzhu leaves from the Qinling Mountains. The inner walls are lined with hundreds of layers of Sago paper and white cotton cloth, treated with materials like blood and lime, and sealed with beeswax and boiled rapeseed oil (5). This unique storage method gives Feng-flavor Baijiu its distinctive flavor and quality (6–8), as shown in Figure 1.

The impact of Mare Nectaris on baijiu has been the subject of some recent studies. According to research by Liu and others, baijiu aged in Mare Nectaris has physicochemical indices that are very different from those aged in ceramic jars. The oxygen permeability of Mare Nectaris is higher than that of ceramic jars because of the production method, the use of lime, and some biological ingredients. As a result, the pH of the baijiu rises and the total acids fall, exhibiting the opposite tendency of ceramic jars (9). Feng and colleagues discovered that baijiu may produce a distinct “Mare Nectaris flavor” when stored in Mare Nectaris. The sensory analysis of the “Mare Nectaris flavor” was conducted using quantitative descriptive analysis (QDA) and check-all-that-apply (CATA). The results showed that aged baijiu stored in Mare Nectaris had distinctive aroma characteristics including woodiness and spiciness (10). Combining foodomics and multivariate analysis, Jia and colleagues noted that the long-term interaction between the baijiu and container materials (beeswax, animal blood, etc.) is the primary cause of the honey-like aroma of Feng-flavor Baijiu that has been kept in Mare Nectaris for 17 years. These interactions not only encourage the synthesis of aromatic compounds but also improve their stability through material migration and metal ion-catalyzed processes (11).

The above research has systematically revealed the molecular formation mechanism of Feng-flavor Baijiu aroma, laying a theoretical foundation for quality-oriented regulation. However, the potential regulatory role of the storage vessel itself—the Mare Nectaris—on the aging process remains unexplored, and there is a lack of quantitative comparisons of their effects on the chemical composition and sensory characteristics of the baijiu. Repeated use causes the Mare Nectaris's wood, rattan matrix, or internal blood layer glue coating to continuously shrink and degrade chemically. Over time, these changes cause gradual changes in micro-oxygen permeability, adsorption/release properties, and interfacial reaction activity. These changes are likely to change the acid-alcohol-ester balance as well as the release or transformation of flavor compounds in the baijiu, which will ultimately affect the final flavor. Thus, a crucial stage in finishing research on Mare Nectaris aging is assessing how age affects the variations in the main volatile flavor constituents and sensory aspects of baijiu.

This study concentrated on the primary variable of Mare Nectaris life in order to investigate the impact of this species on the body flavor and sensory quality of Baijiu. Significant variations and internal correlations in the flavor substance composition, sensory attributes, and overall quality shaping were methodically examined by a thorough examination of the preserved baijiu from various service years, as shown in Graphical Abstract. The objectives of this research were to highlight the special role that Mare Nectaris' service life plays in the aging process of the baibjiu as well as to offer theoretical underpinnings and useful advice for the scientific management of Mare Nectaris and the accurate regulation of Baijiu quality.

## 2 Materials and methods

### 2.1 Baijiu samples

**Figure 2:**
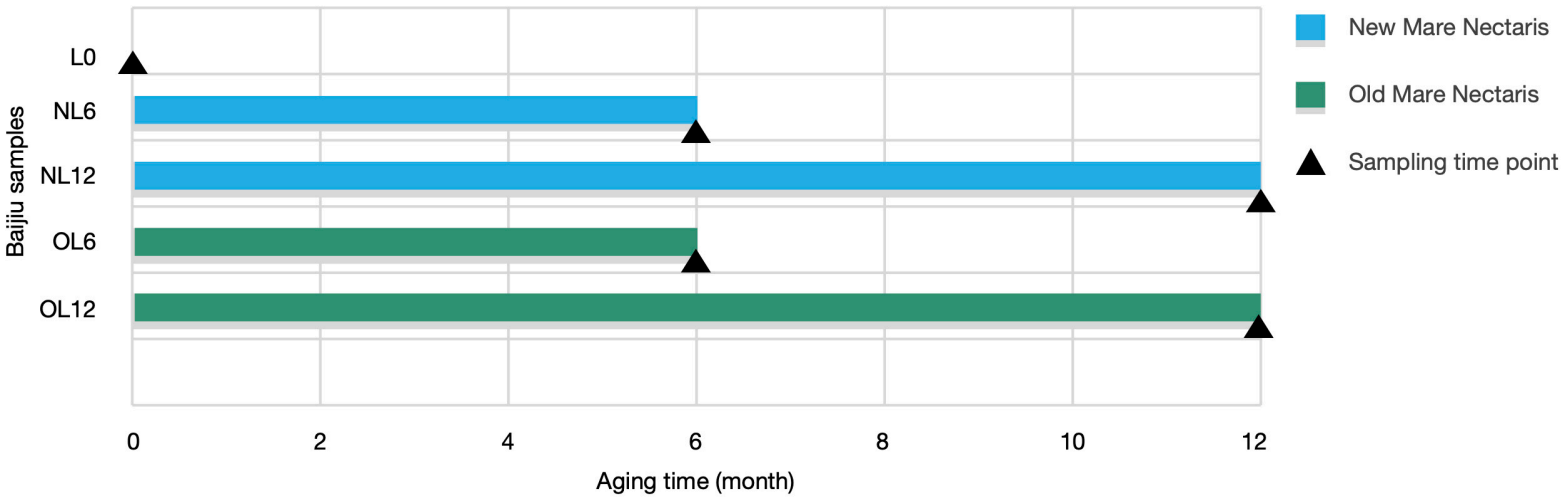
Presents the sampling method. Samples stored in new and old Mare Nectaris were designated “NL” and “OL.” The number after the letter indicates the storage time in months.

Feng-flavor base Baijiu was stored in new (< 5 years) and old (>10 years) Mare Nectaris for 6 and 12 months. Samples collected include L0 (control, no storage), NL6 and NL12 (new Mare Nectaris), and OL6 and OL12 (old Mare Nectaris). All samples were provided by Liulin Baijiu Industry Co., Ltd. (Baoji, China), and produced using the same raw materials and brewing process and analyzed after storage. Sampling methods are illustrated in Figure 2.

### 2.2 Reagents

Sinopharm Chemical Reagent Co., Ltd. (Shanghai, China) supplied analytical-grade sodium chloride and ether. Sigma Aldrich Chemical Company was the supplier of additional chemicals (GC grade, ≥95% purity). The National Center for Analysis and Testing of Non-ferrous Metals and Electronic Materials supplied the Ca standard solution (1,000 μg/ml), and purified water was acquired from the Milli-Q system (Millipore, Bedford, USA).

### 2.3 Sensory analysis methods

#### 2.3.1 Panel members

The sensory study was carried out by two different panels: the expert panel and the laboratory panel. The Industry Panel consisted of five expert tasters from the China National Research Institute of Food and Fermentation Industries, three of whom were men and two of whom were women, ranging in age from 28 to 48. They all had over 3 years of expertise in sensory tasting, regularly participated in sensory analysis investigations, and were national Baijiu assessors.

Ten assessors, five male and five female, aged 26–50, in good health and free of bad habits, with over 2 years of experience in sensory tasting, regular training in spirits sensory tasting, and a concealed test accuracy rate of over 80% in aroma judgment, make up the laboratory team. All assessors are able to identify and explain sample differences.

#### 2.3.2 Descriptive analysis of Baijiu storage samples

The laboratory team finished determining the sensory descriptions. The samples were placed in clean Baijiu cups labeled with a random code, and team members independently sniffed and tasted them in a different section of the professional tasting room at room temperature (21 ± 1 °C). Each of the three rounds of the experiment included a cup of 20 ml of three different kinds of Baijiu samples, and each round was assessed for 30 min. Between rounds, you must gargle with mineral water for a duration of 15 min. After the experiment, the participants were asked to enumerate particular characteristics of their emotions, such as taste, texture, and scent. (Description requirements: the description language cannot contain hedonistic or quantitative terms like “delicious” or “suitable”). For descriptive analysis, panelists were asked to enumerate representative scent features. Eleven characteristics of scent, taste, and style—“honey,” “fruity,” “floral,” “MareNectaris-aroma,” “Chen,” “lime,” “sweet,” “astringent,” “bloody,” “clean,” and “coordinate”—were chosen for review following the earlier investigation. Samples are supplied one after the other, separated by 3 min. Following three evaluations of each sample, the sensory score is averaged and then presented as a radarmap.

### 2.4 Quantitative analysis of volatile compounds

#### 2.4.1 Gas chromatography-flame ionization detection analysis

Through the use of gas chromatography-flame ionization detection (GC-FID), the volatile chemicals of the samples were precisely measured. Ethanol (HPLC) was added to the Baijiu sample until the volume fraction of ethanol reached 70.0% because varying water content would alter the detection results. 10 μl of internal standard (IS1, IS2) was added to 1.0 ml of treated Baijiu samples. Then, using a GC-FID fitted with a CP-WAX 57 CB capillary column (50 m × 0.25 mm × 0.2 μm; J&W Scientific Corporation, USA), the mixed samples were examined. In split mode (10:1), the samples (1.0 μl) were injected into the GC mentioned above. Air flow rate was 450 ml/min; detector temperature was 270 °C; injector temperature was 240 °C; carrier gas was nitrogen (99.999%), with a flow rate of 1 ml/min; and hydrogen (H_2_), with a flow rate of 45 ml/min. The temperature was raised from 35 to 60 °C at 4 °C/min and sustained for 2 min, then to 110 °C at 6 °C/min and maintained for 3 min, and lastly to 205 °C at 6 °C/min and maintained for 13 min. Every experiment was carried out three times.

#### 2.4.2 Ion chromatography analysis

Lactic acid, acetic acid, propionic acid, formic acid, and other somewhat high-content compounds were among the organic acids identified in the samples after they had been diluted 50 times with pure water. 250 mm x 4 mm Impact AS11-HC separation column, 50 mm x 4 mm Ionpac AG11-HC protection column, The conductivity detector observed the eluent's flow rate of 1.0 ml/min. 25 μl was the injection volume. The following was the KOH cleaning program: KOH concentration ranged from 0 to 16 min at 1.1 mmol/L. The concentration of KOH rose to 16.5 mmol/L at 17–29 min and to 20.0 mmol/L at 29–35 min. The KOH content rose to 35 mmol/L at 35–39 min and remained steady for 2 min. The KOH concentration rose to 50.0 mmol/L between 41 and 47 min, dropped to 1.1 mmol/L at 47.1 min, and then stayed constant until 59 min.

### 2.5 Odor activity values (OAVs)

The OAV, which is computed by dividing the compound concentration (C) by its odor threshold (OT), was used to assess the contribution of each constituent to aroma. OAV ≥1 compounds were thought to play a major role in the aroma characteristics (12):


$$\begin{array}{l} \text{O}\text{A}\text{V}=\frac{\text{C}}{\text{T}} \end{array}$$

### 2.6 Metal ion quantification and pH analysis

The GBC Avanta Atomic Absorption Spectrometer and the PHS-25 digital display benchtop acidity meter are both advanced instruments, with the former being used for elemental analysis and the latter for measuring acidity, and the PHS-25 is produced by Remagnetics in Shanghai, China.

### 2.7 Statistical analysis

For triple analyses, the results are displayed as mean ± standard deviation. Duncan's test and one-way ANOVA were used to identify significant differences (*p* < 0.05). The variations between base Baijiu under various storage conditions were examined using PCA. Using OriginPro2018 (OriginLab, Northampton, MA) and SPSS (version 26.0) for MAC, the connection between base Baijiu, volatile chemicals, and sensory qualities under various storage settings was examined. The Chiplot online analytic tool (https://www.chiplot.online/index.html) was used to plot radar maps and cluster heatmaps. Using the Hiplot online analytic platform (https://hiplot.com.cn/home/index.html), boxplots, bar charts, and pie charts were plotted.

## 3 Results and discussion

### 3.1 Quantitative and descriptive sensory analysis (QDA)

**Figure 3:**
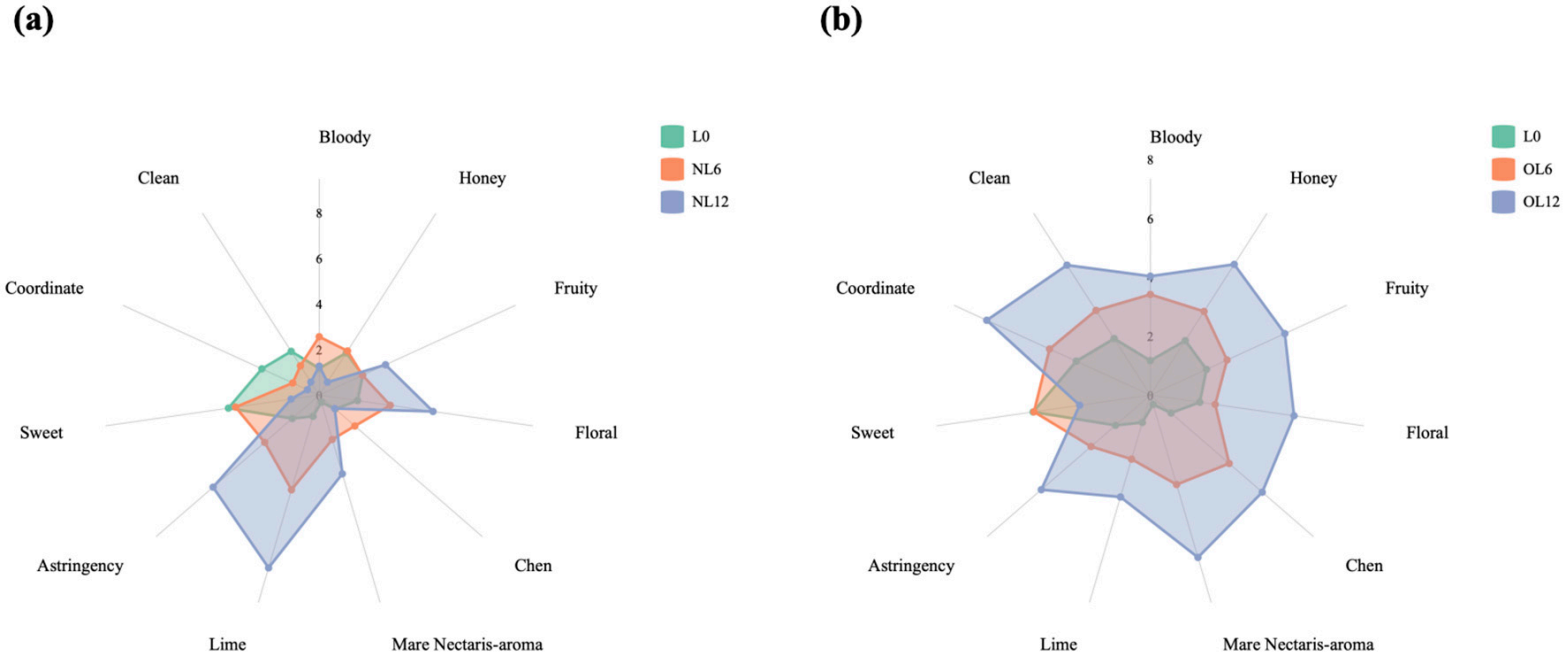
Sensory difference of Feng-flavor Baijiu stored in different Mare Nectaris. Samples stored in new **(a)** and old **(b)** Mare Nectaris were designated “NL” and “OL” respectively. The number after the letter indicates the storage time in months.

The storage of Mare Nectaris brings different sensory characteristics to the base Baijiu. A total of 11 sensory descriptors were selected, including five aromas (honey, fruity, floral, Chen, and Mare Nectaris-aroma), four flavors (lime, bloody, sweet, and astringent), and two tastes (coordinate and clean). The data obtained were plotted in the radar maps (Figure 3).

The findings demonstrated that the Mare Nectaris storage had varying impacts on the basic Baijiu and that, depending on the conditions, the Mare Nectaris had significant effects on one's senses. The positive sensory qualities of OL6 (3.38), fruity (2.87), floral (2.23), Chen (3.55), and Mare Nectaris-aroma (3.17) were improved when the Baijiu samples were kept in the old Mare Nectaris, while the negative qualities of bloody (3.42), lime (2.27), and astringency (2.67) were marginally improved. However, the Baijiu has a deeper flavor than L0 because of the favorable sensory qualities brought about by preservation, hence the coordinate (3.77) is stronger than L0 (2.77); The positive sensory qualities of OL12, such as honey (5.28), fruity (5.03), floral (4.94), Chen (5.04), and Mare Nectaris-aroma (5.75), became more noticeable as the storage period was extended. This suggests that the beneficial effects of old Mare Nectaris on Baijiu bodies were primarily evident in these five aspects. The coordination sense (6.12) and clean sense (5.25) had the highest scores, despite the lime taste (3.61), suggesting that the Baijiu body was rich and balanced at this time. As a result, the storage of old Mare Nectaris enhanced the quality of the Baijiu sample, and the overall style was more typical.

While the aroma (5.05), lime (7.9), and astringency (6.17) were prominent after 12 months of storage, the sensory scores of NL6 for aroma (3.15), lime (4.33), and astringency (3.17) were higher than L0 during the storage process of new Mare Nectaris. Additionally, the enhancement of lime and astringency decreased body coordination (1.28), and clean (1.53). Analysis NL12 may be the result of the strong lime flavor masking the Mare Nectaris-aroma and fruit, including the Mare Nectaris wall coating, which may have been brought on by the pig blood smell, but did not mask the floral, which is relatively prominent (5.05). Fruity (3.2) and Mare Nectaris-aroma (3.6), with coordination (0.57) and clean (0.68) being the lowest among these.

A thorough investigation revealed that the Baijiu body of both the old and new Mare Nectaris stored had varying degrees of the unfavorable attribute lime taste. It is hypothesized that this taste is connected to the quick lime (an alkaline substance) that is present in the inner wall coating of the Mare Nectaris. Calcium hydroxide is created when quick lime and water combine, neutralizing the organic acids in the Mare Nectaris and lowering the overall acidity. Simultaneously, as storage migrates, the amount of calcium ions the lime brings will rise.

**Figure 4:**
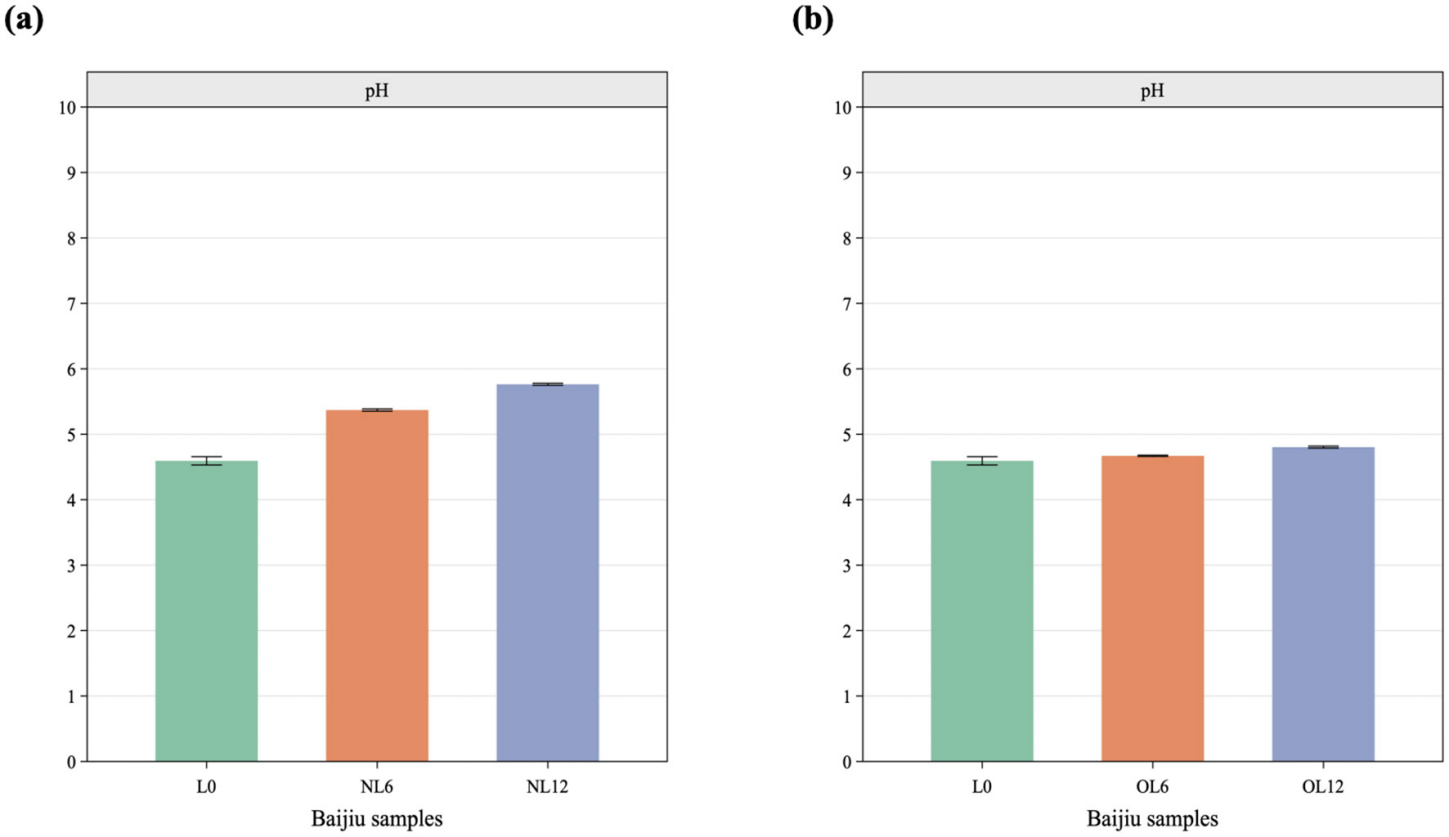
pH values detected in Feng-flavor Baijiu with different Mare Nectaris. Samples stored in new **(a)** and old **(b)** Mare Nectaris were designated “NL” and “OL” respectively. The number after the letter indicates the storage time in months.

**Figure 5:**
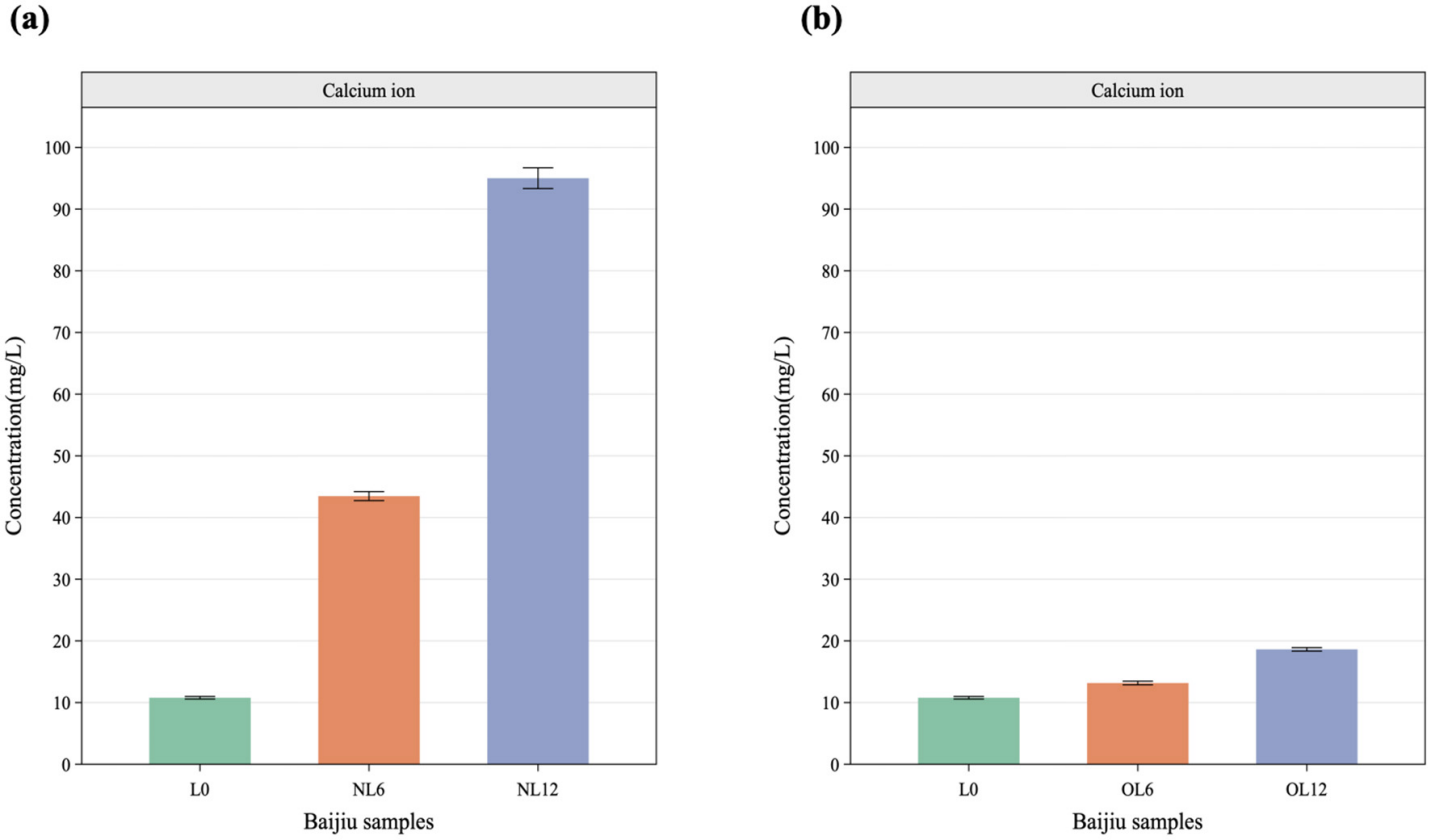
Analysis of calcium ions in Feng-flavor Baijiu with different Mare Nectaris. Samples stored in new **(a)** and old **(b)** Mare Nectaris were designated “NL” and “OL” respectively. The number after the letter indicates the storage time in months.

**Figure 6:**
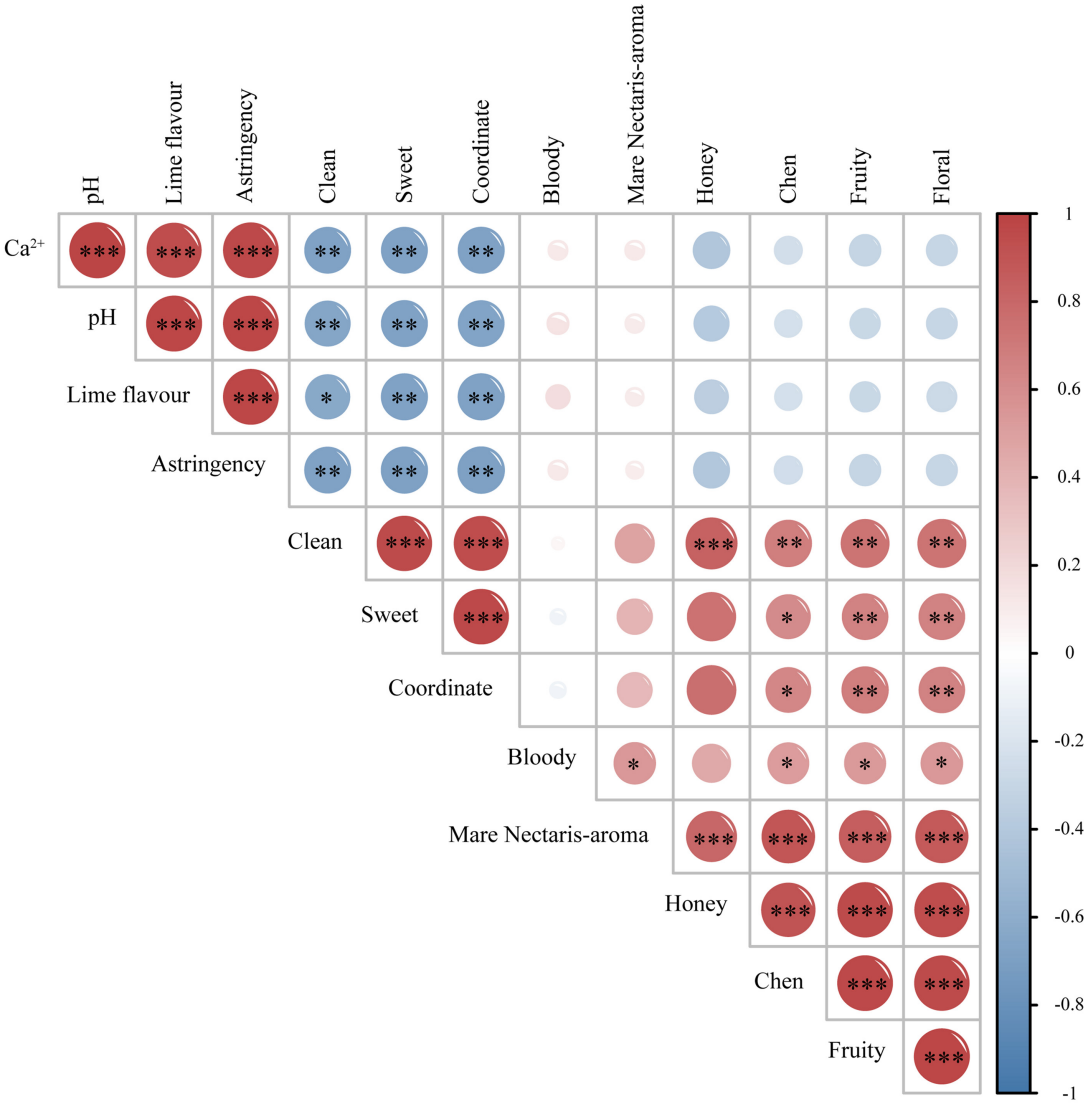
Correlation analysis of Sensory, pH, and metal ions in Feng-flavor Baijiu stored in new and old Mare Nectaris (sample correlation heat map; *P < 0.05, **P < 0.01, ***P < 0.001; red indicates positive correlation, blue indicates negative correlation).

The samples' pH and calcium ions were measured, as seen in Figures 4, 5. After a year of storage in the old Mare Nectaris samples, the pH of the newly made Baijiu L0 rose from 4.63 to 4.79 and the calcium ion content rose from 10.78 to 18.62 mg/L. The new Mare Nectaris had a pH of 5.76 instead of 4.63, a calcium ion content of 95.01 mg/L, and a calcium ion dissolution rate that was 10.82 times greater than that of the old Mare Nectaris. The calcium concentration was proportionate to the storage period within a year. Figure 6, the correlation analysis shows that sensory evaluation is mostly split into two groups. The left branch is floral, fruity, Chen, Mare Nectaris-aroma, bloody, coordinate, sweet, and clean, whereas the right branch is primarily made up of pH, calcium ions, the negative assessment of lime, and astringency. The flavor and astringency of lime were substantially positively connected with the pH and calcium ions of the Baijiu samples.

The inner wall coating of the Mare Nectaris was the cause of the lime flavor in the samples, and the various levels of astringency in the samples were rated adversely, according to the assessment results shown in the radar map above. The aromas of honey, floral, fruit, and Chen were significantly positively correlated with the aroma of Mare Nectaris. This indicated that the storage of Mare Nectaris not only produced the aroma of Mare Nectaris but also honey, floral, fruit, and Chen, which contributed to the rich flavor of the baijiu body.

### 3.2 Analysis of major volatile compounds

**Figure 7:**
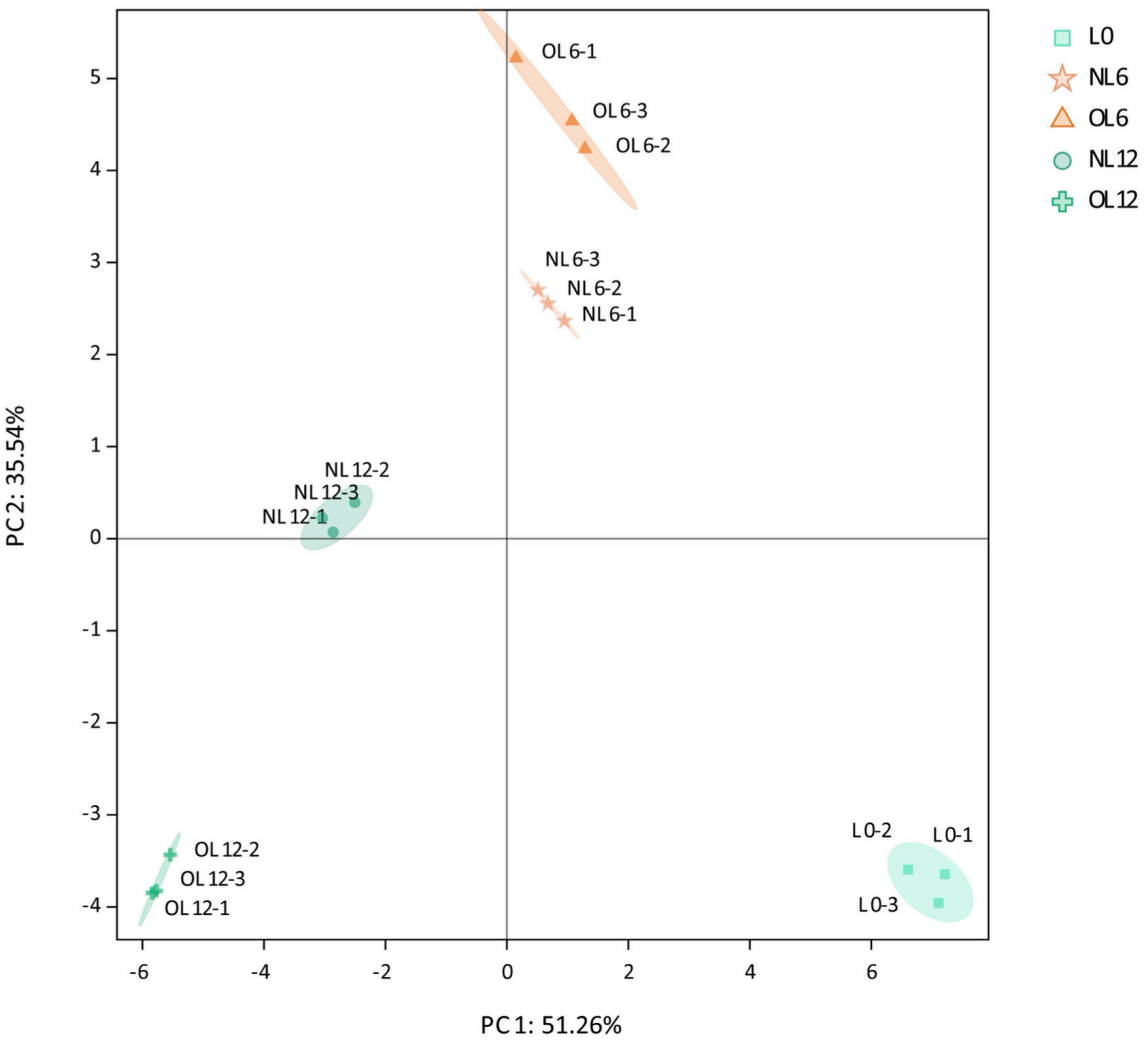
PCA analysis of flavor compounds in Feng-flavor Baijiu stored in different Mare Nectaris. Samples stored in new and old Mare Nectaris were designated “NL” and “OL,” respectively. The number after the letter indicates the storage time in months.

The samples of base baijiu, old Mare Nectaris, and new Mare Nectaris contained a total of forty-four volatile compounds. Twelve alcohols, nine carbonyls, nineteen esters, and four acids were among the compounds. Principal component analysis (PCA) was performed on the quantitative data of the three sample categories. The findings in Figure 7 indicated that two PCs accounted for 80.6% of the overall variation (PC2 = 35.5%, PC1 = 51.26%). The unstored baijiu was easily separated from the samples in the new and old Mare Nectaris in the PCA scoring plot. This implies that the baijiu's volatile chemical composition has changed significantly as a result of container storage.

PC1 was intimately linked to the shift in volatile compounds in baijiu brought on by storage time, as seen by the samples kept in the new Mare Nectaris, which were ordered from right to left based on storage time (June to December). The baijiu samples that were kept in the new Mare Nectaris for 6 months were found to be in P1's positive direction and P2's negative direction. The old baijiu samples were in the negative direction of PC1 and PC2, whereas the fresh baijiu samples were dispersed in the positive direction of PC2 and the negative direction of PC1 after a year of storage.

This showed that the distance between OL6 and OL12 in the PC1 direction was longer than that between NL6 and NL12, and that the storage conditions of samples had distinct impacts on volatile compounds in the latter storage period. According to the data, the old Mare Nectaris had a greater impact on volatile flavor compounds over the course of storage than the newer Mare Nectaris.

**Figure 8:**
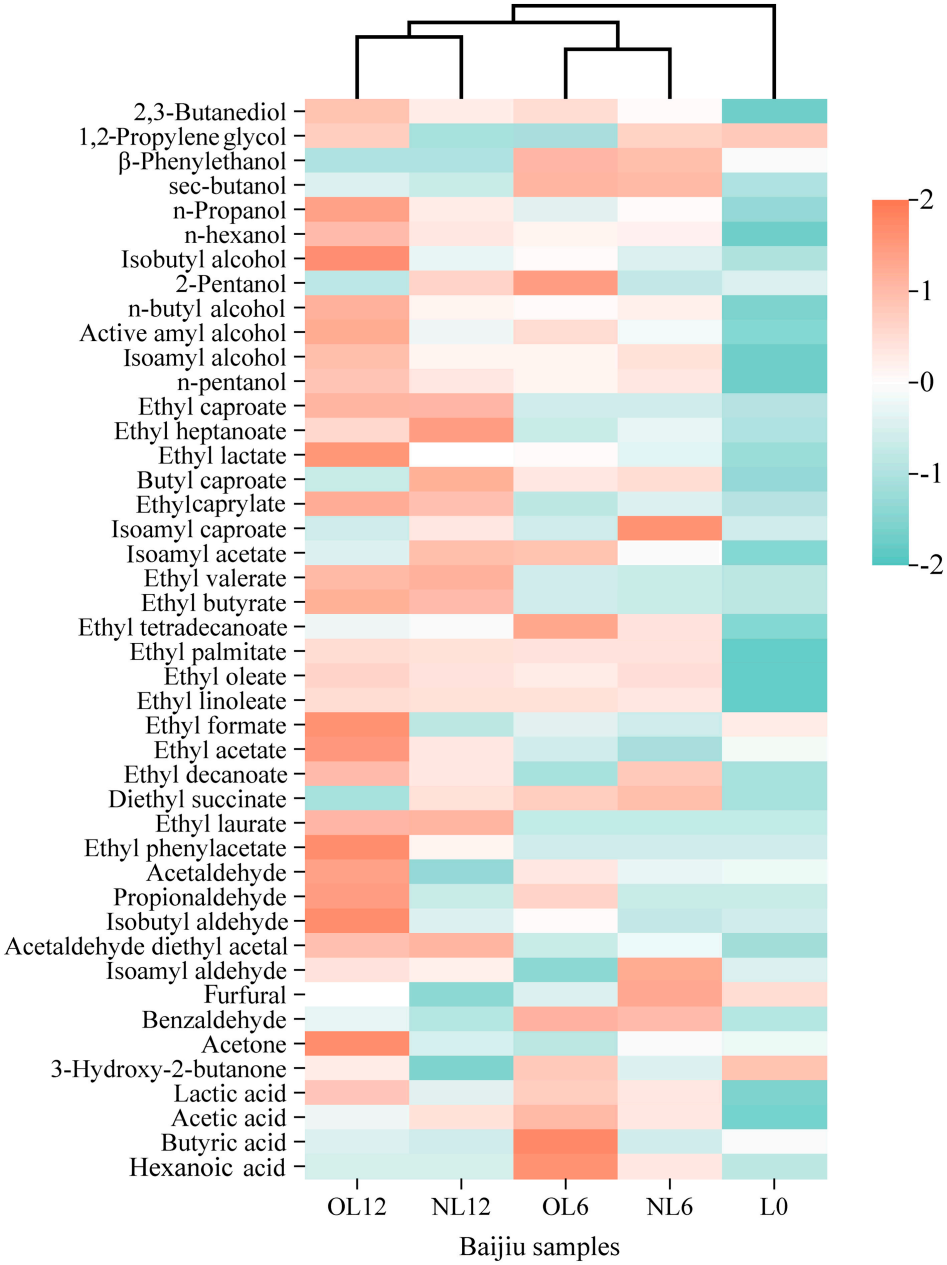
Cluster analysis heat map. Samples stored in new and old Mare Nectaris were designated “NL” and “OL”. The number after the letter indicates the storage time in months.

Moreover, it is evident from Figure 8 heat map of quantitative data of all volatile compounds in samples that all samples are grouped and stratified into two groups: L0 and NL6, OL6, NL12, and OL12. This suggests that the volatile compounds in unstored baijiu differ significantly from those in stored samples. Two additional classes were created from the left main branch: NL6 and OL6 and NL12 and OL12.

**Figure 9:**
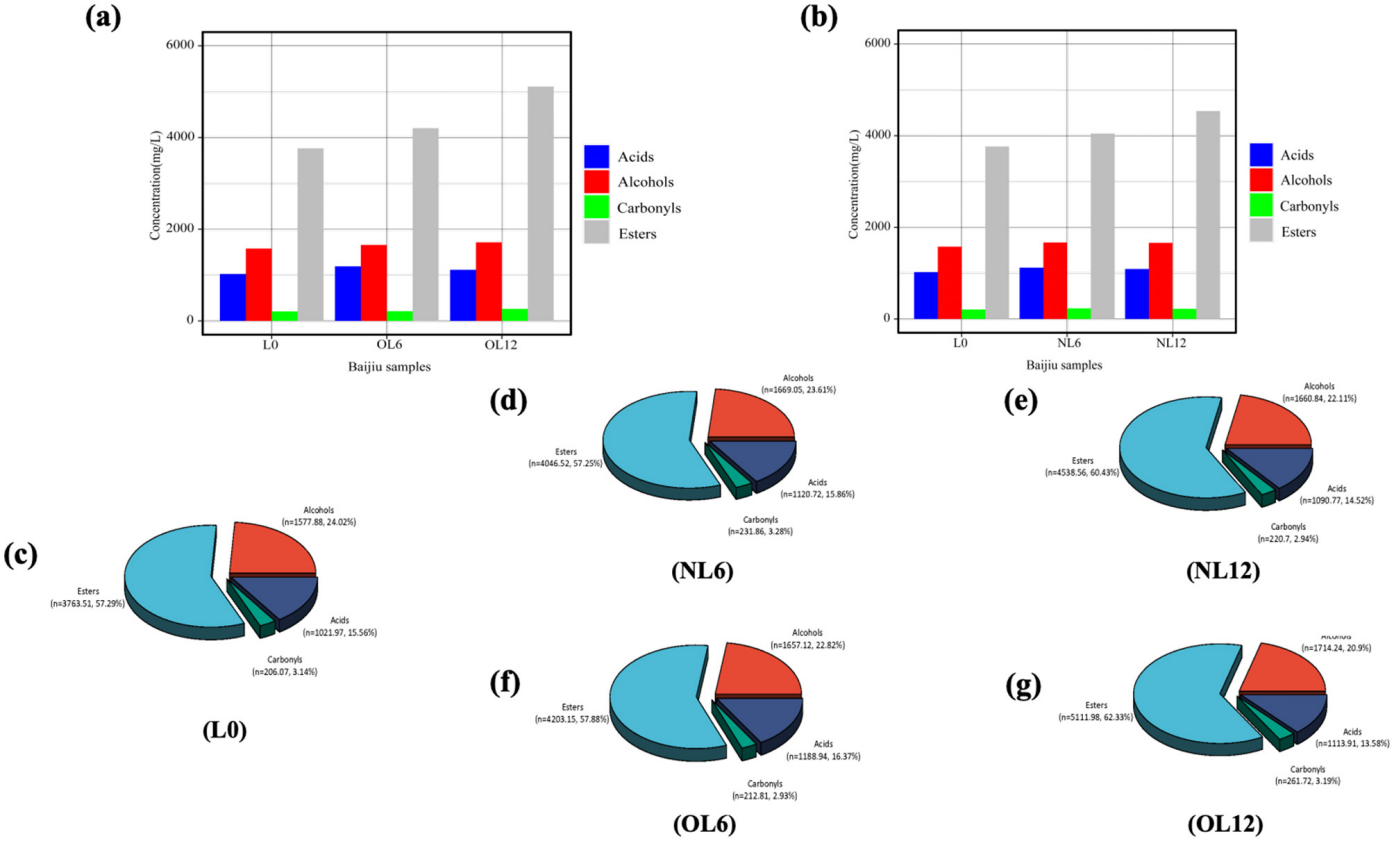
Contents of flavor compounds in Baijiu stored in old **(a)** and new **(b)** Mare Nectaris; pie charts showing the relative proportions of the main flavor compounds in untreated **(c)** and in samples stored for different periods in new **(d, e)** and old **(f, g)** old Mare Nectaris.

The difference in volatile flavor compound content between the original samples and the preserved samples, as well as changes over storage time, may be shown in Figure 9 together with Table 1. In every sample, esters were the most prevalent (57.2%−62.3%). Acids (13.6%−16.3%), carbonyls (2.9%−3.2%), and alcohols (20.9%−24.0%) come next. The samples included comparable volatile compounds in varying amounts, as indicated in Table 1.

**Table 1:** Quantitative results of volatile compounds in Feng-flavor Baijiu samples.

**15.6-2.2,-1.3498ptCompounds**	**L0**	**NL6**	**NL12**	**OL6**	**OL12**
**Alcohols**					
2,3-Butanediol	20.00 ± 0.48^d^	23.86 ± 0.30^c^	24.31 ± 0.59^bc^	24.91 ± 0.52^ab^	25.78 ± 0.54^a^
1,2-Propylene glycol	16.46 ± 0.31^a^	16.13 ± 0.33^a^	11.52 ± 0.21^b^	11.45 ± 0.19^b^	16.28 ± 0.33^a^
β-phenyl ethanol	1.03 ± 0.05^c^	2.91 ± 0.04^b^	ND^d^	3.11 ± 0.07^a^	ND^d^
sec-butanol	47.63 ± 0.69^b^	50.82 ± 1.15^a^	48.11 ± 1.06^b^	50.91 ± 1.24^a^	48.48 ± 1.22^b^
n-Propanol	495.94 ± 8.55^b^	513.28 ± 12.71^a^	516.05 ± 10.78^a^	507.92 ± 9.70^b^	530.54 ± 10.41^a^
n-hexanol	131.33 ± 3.64^c^	146.88 ± 3.79^b^	148.39 ± 3.88^ab^	146.72 ± 2.91^b^	153.96 ± 4.12^a^
Isobutyl alcohol	101.06 ± 2.09^a^	101.50 ± 1.40^a^	101.67 ± 1.41^a^	101.96 ± 1.88^a^	103.31 ± 2.56^a^
2-Pentanol	5.13 ± 0.13^c^	5.03 ± 0.11^c^	5.54 ± 0.08^b^	5.82 ± 0.14^a^	5.00 ± 0.11^c^
N-butyl alcohol	450.72 ± 7.09^b^	478.69 ± 10.87^a^	477.93 ± 6.61^a^	476.23 ± 12.72^a^	494.13 ± 9.63^a^
Active amyl alcohol	38.51 ± 1.09^c^	40.29 ± 0.86^b^	40.21 ± 0.61^b^	41.23 ± 0.85^ab^	42.23 ± 0.81^a^
Isoamyl alcohol	245.50 ± 4.54^b^	263.52 ± 5.86^a^	260.95 ± 4.54^a^	260.91 ± 4.33^a^	267.93 ± 7.51^a^
15.6-2.2,-1.3498ptn-pentanol	24.19 ± 0.39^b^	26.10 ± 0.43^a^	26.10 ± 0.64^a^	25.89 ± 0.34^a^	26.55 ± 0.52^a^
**Esters**					
Ethyl caproate	580.99 ± 12.23^c^	623.67 ± 15.16^b^	814.77 ± 14.17^a^	617.45 ± 14.87^b^	818.16 ± 14.38^a^
Ethyl heptanoate	15.59 ± 0.31^e^	17.48 ± 0.34^c^	22.12 ± 0.51^a^	16.41 ± 0.46^d^	19.89 ± 0.34^b^
Ethyl lactate	1,696.19 ± 23.33^d^	1,930.12 ± 40.24^c^	2,020.73 ± 34.98^b^	2,033.74 ± 29.64^b^	2,445.41 ± 40.84^a^
Butyl caproate	ND^e^	5.53 ± 0.14^b^	7.59 ± 0.13^a^	5.01 ± 0.11^c^	1.80 ± 0.03^d^
Ethyl caprylate	17.70 ± 0.37^d^	19.50 ± 0.41^c^	24.59 ± 0.43^b^	18.14 ± 0.36^d^	25.80 ± 0.53^a^
Isoamyl caproate	ND^c^	1.59 ± 0.02^a^	0.72 ± 0.01^b^	ND^c^	ND^c^
Isoamyl acetate	ND^d^	2.77 ± 0.06^b^	4.56 ± 0.09^a^	4.44 ± 0.10^a^	1.96 ± 0.03^c^
Ethyl valerate	37.18 ± 0.96^c^	38.19 ± 0.88^bc^	47.74 ± 0.54^a^	38.74 ± 0.96^b^	47.09 ± 0.64^a^
Ethyl butyrate	94.16 ± 1.89^b^	97.13 ± 1.88^b^	119.91 ± 2.45^a^	97.47 ± 2.03^b^	122.45 ± 2.49^a^
Ethyl tetradecanoate	ND^e^	3.09 ± 0.07^b^	2.27 ± 0.04^a^	4.58 ± 0.06^c^	2.04 ± 0.04^d^
Ethyl palmitate	ND^c^	39.95 ± 0.80^b^	40.01 ± 0.59^b^	39.57 ± 0.47^b^	41.38 ± 0.88^a^
Ethyl oleate	ND^d^	26.93 ± 0.62^b^	26.39 ± 0.69^b^	24.95 ± 0.39^c^	28.81 ± 0.54^a^
Ethyl linoleate	ND^c^	30.06 ± 0.70^b^	31.37 ± 0.62^a^	31.30 ± 0.76^a^	32.46 ± 0.78^a^
Ethyl formate	15.17 ± 0.38^b^	11.25 ± 0.27^d^	10.35 ± 0.18^e^	12.35 ± 0.20^c^	20.75 ± 0.46^a^
Ethyl acetate	1,306.4 ± 19.63^c^	1,195.77 ± 26.00^e^	1,361.23 ± 19.11^b^	1,257.43 ± 23.11^d^	1,498.78 ± 40.84^a^
Ethyl decanoate	ND^d^	1.72 ± 0.12^b^	1.27 ± 0.02^c^	ND^d^	1.93 ± 0.03^a^
Diethyl succinate	ND^d^	1.67 ± 0.03^a^	1.25 ± 0.02^c^	1.50 ± 0.03^b^	ND^d^
Ethyl laurate	ND^c^	ND^c^	0.83 ± 0.01^a^	ND^c^	0.81 ± 0.01^b^
15.6-2.2,-1.3498ptEthyl phenylacetate	ND^c^	ND^c^	0.77 ± 0.01^b^	ND^c^	2.38 ± 0.04^a^
**Carbonyls**					
Acetaldehyde	86.99 ± 1.36^c^	86.26 ± 2.09^c^	75.05 ± 1.95^d^	92.44 ± 1.98^b^	103.47 ± 1.41^a^
Propionaldehyde	ND^c^	ND^c^	ND^c^	1.23 ± 0.02^b^	1.97 ± 0.05^a^
Isobutyl aldehyde	5.19 ± 0.09^d^	4.93 ± 0.08^e^	5.55 ± 0.12^c^	6.41 ± 0.15^b^	9.69 ± 0.20^a^
Acetaldehyde diethyl acetal	72.95 ± 1.13^d^	84.34 ± 1.44^b^	99.31 ± 1.90^a^	78.37 ± 1.21^c^	97.44 ± 2.54^a^
Isoamyl aldehyde	27.69 ± 0.54^d^	41.37 ± 0.79^a^	33.02 ± 0.73^c^	20.60 ± 0.33^e^	34.72 ± 0.76^b^
Furfural	2.23 ± 0.03^b^	3.14 ± 0.03^a^	ND^e^	1.11 ± 0.03^d^	1.61 ± 0.04^c^
Benzaldehyde	ND^d^	2.24 ± 0.04^b^	ND^d^	2.40 ± 0.02^a^	0.68 ± 0.01^c^
Acetone	8.03 ± 0.20^bc^	8.17 ± 0.13^b^	7.74 ± 0.11^c^	7.37 ± 0.15^d^	9.90 ± 0.18^a^
15.6-2.2,-1.3498pt3-Hydroxy-2-butanone	2.97 ± 0.06^a^	1.38 ± 0.03^d^	ND^e^	2.83 ± 0.05^b^	2.21 ± 0.04^c^
**Acids**					
Lactic acid	124.55 ± 2.66^d^	177.90 ± 4.62^b^	156.98 ± 3.70^c^	188.31 ± 5.11^a^	190.49 ± 2.34^a^
Acetic acid	533.38 ± 12.99^c^	570.99 ± 11.14^ab^	572.79 ± 9.26^ab^	585.06 ± 12.60^a^	560.42 ± 11.09^b^
Butyric acid	129.50 ± 2.21^b^	123.85 ± 3.34^c^	123.13 ± 2.31^c^	152.05 ± 2.24^a^	125.18 ± 1.41^bc^
Hexanoic acid	234.52 ± 3.90^c^	247.96 ± 4.45^b^	237.86 ± 4.08^c^	263.51 ± 7.14^a^	237.79 ± 4.37^c^

Concentration is the mean ± SD of triplicate (mg/L).

Name: L0, base Baijiu; NL, new Mare Nectaris; OL, old Mare Nectaris.

ND, not detected.

Different letters in the same row indicate significant differences (*p* ≤ 0.05).

#### 3.2.1 Changes in major alcohol-derived flavor compounds

**Figure 10:**
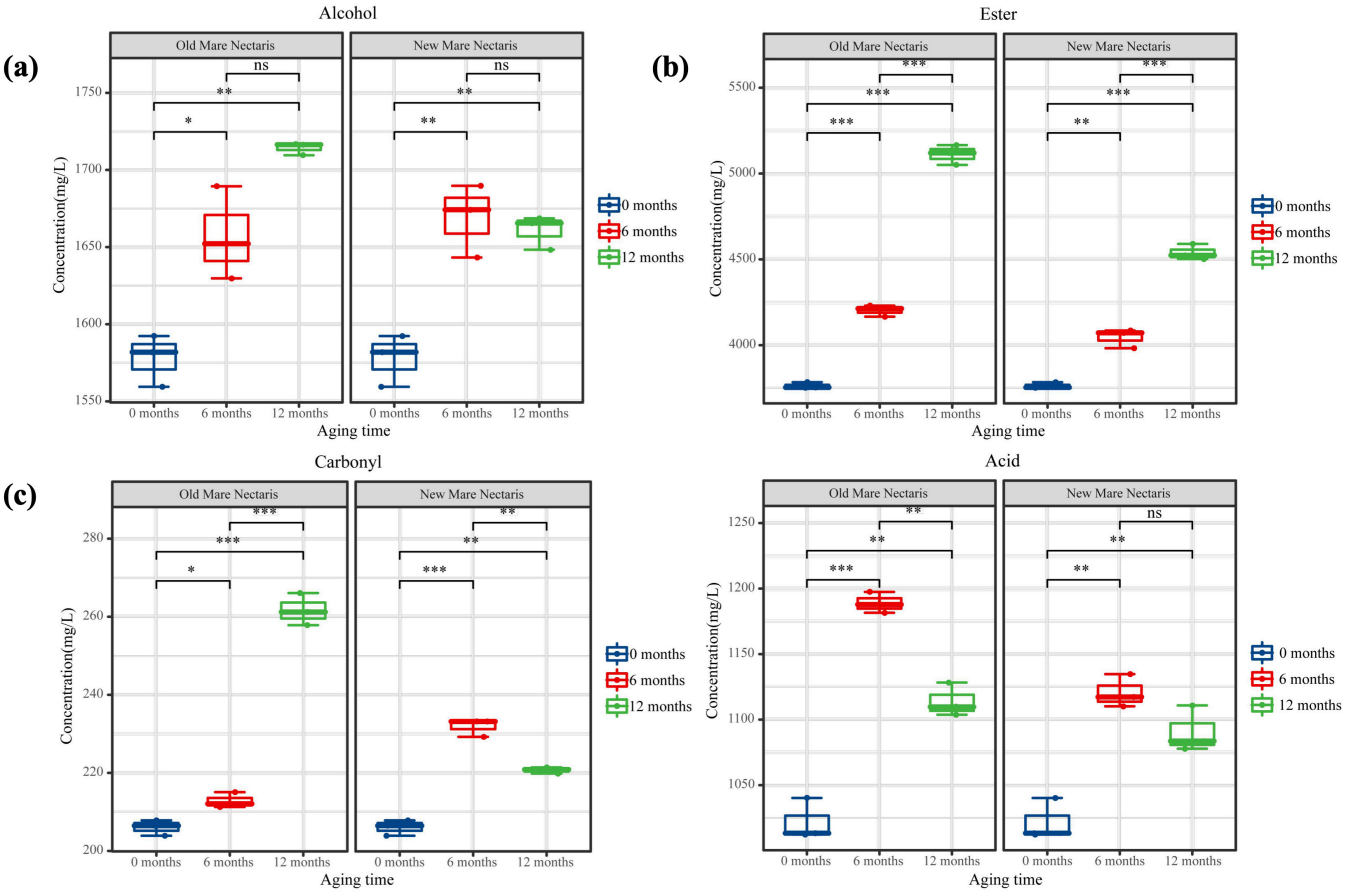
Content of alcohols **(a)**, esters **(b)**, carbonyls **(c)**, and acids **(d)** in Baijiu stored in Mare Nectaris. Samples stored in new and old Mare Nectaris were designated “NL” and “OL,” respectively; the number after the letter indicates storage time in months. Significance: ns (not significant), * (significant, *p* < 0.05), ** (highly significant, *p* < 0.01), and *** (very highly significant, *p* < 0.001).

Alcohols are the primary source of mellow sweetness and aroma and the precursor of esters in Baijiu (13). The boiling points of alcohols are low, making them easy to volatilize, which enriches the flavor of Baijiu (14). The major alcohol levels in Feng-flavor Baijiu rose over storage, as seen in Figure 10. In addition to ethanol, the most prevalent substances were isoamyl alcohol (fruity, almond-like), n-propanol (fruity, grassy), and n-butanol (floral), which combined accounted for 75.5% of the total alcohol fraction. In line with Huang Ting's findings, the concentrations of isoamyl alcohol and n-propanol increased throughout storage time (15). The reversible hydrolysis can result in a net increase in some alcohols because this behavior is related to the esterification/hydrolysis equilibrium that defines baijiu maturation: alcohols are first oxidized to aldehydes and acids, which then combine with remaining alcohols to form esters; these esters are then hydrolyzed to regenerate acids and alcohols. Reductive chemicals (H_2_S, thiols) generated during the breakdown of egg-white proteins in the Mare Nectaris layer, which convert aldehydes back to their corresponding alcohols, are likely responsible for the extra rise. In fact, the “Mare Nectaris-aroma” of baijiu occasionally has a subtle “egg-white” off-note. After being stored in the new Mare Nectaris, the content of 2,3-butanediol rose from 19.99 mg L^−1^ in L0 to 23.86–25.78 mg L^−1^, which would have added a sharp, slightly bitter edge (16). The honeyed character frequently found in Mare Nectaris-aged baijiu is therefore probably related to this higher β-phenylethanol level, which is presumably leached from the beeswax layer applied to the inner wall. β-phenylethanol, which conveys rose or honey-like notes and is a key aroma contributor in rice-aroma baijiu (26), also increased after 6 months in both new and old Mare Nectaris. The relative percentage of these major alcohols in the total volatiles, however, decreased over time, going from 24.0 to 22.1% in new Mare Nectaris and to 20.9% in old Mare Nectaris, as Figure 9 shows. Thus, despite the above-mentioned absolute increases, the decline was more noticeable in the older containers, demonstrating their stronger net removal or modification of alcohols.

#### 3.2.2 Changes in major ester-derived flavor compounds

Esters lengthen the aftertaste and give Baijiu rich fruity and floral aromas. Esters make up 57.3% of 0's total, as seen in Figures 9, 10. The percentage increased to 60.4 and 62.3%, respectively, after a year of storage in new and old Mare Nectaris. In the first 6 months, the proportions were the same, suggesting that the major alterations happened later. According to Ting et al.'s research, the amount of esters such ethyl caproate and ethyl butyrate rose with storage time. Following preservation in baijiu containers, the amount of ethyl lactate rose, which is in line with Lili et al.'s findings. Ethyl lactate played an important role in the quality of Baijiu (17–19). In the 0–6 and 6–12 month stages, there were notable changes between the new and old Mare Nectaris. Ethyl lactate level rose from 1,696.19 to 1,930.12 mg/L after 6 months of storage in new Mare Nectaris, representing a 13.8% rise rate and a 233.93 mg/L increase. It only climbed by 90.61 mg/L in the next 6 months, at a rate of 4.7%. This means that the first 6 months accounted for 72% of the 12-month growth, and the increase tended to level out in the latter half of the period. During the same time period, the level of the old Mare Nectaris rose from 1,696.19 to 2,033.74 mg/L, representing a 337.55 mg/L rise and a 19.9% increase rate. With an increase of 411.67 mg/L and an increase rate of 20.2%, it continued to rise to 2,445.41 mg/L between 6 and 12 months, exhibiting a “secondary acceleration” feature. The ancient Mare Nectaris grew by 749.22 mg/L overall during the course of the 12-month period, representing an overall rise rate of 44.2%. The aforementioned findings suggest that ethyl lactate buildup is more favorable in older Mare Nectaris. Using old Mare Nectaris and extending the storage period to 12 months are recommended if the amount of ethyl lactate needs to be raised throughout the storage procedure. Butyric acid combines with ethanol to generate ethyl butyrate, and the rise in ethyl butyrate level is accompanied by a reduction in butyric acid, suggesting that esterification processes are dominating at this stage. The production of ethyl butyrate is comparatively unaffected by the Mare Nectaris conditions. After being stored in Mare Nectaris, the amount of ethyl butyrate, ethyl isovalerate, ethyl tetradecanoate, ethyl oleate, and ethyl linoleate in the new baijiu grew; however, the amount changed with time under various conditions. This oscillation might be caused by the new Mare Nectaris' inner covering not yet forming a stable interface, but more research is required to confirm this by monitoring the dynamic changes of the dissolved chemicals.

#### 3.2.3 Changes in major carbonyl-derived flavor compounds

Carbonyl molecules found in baijiu come from a variety of metabolic pathways, including alcohol oxidation, amino acid deamination, and decarboxylation metabolic processes (20). The aroma and flavor of Baijiu are enhanced by carbonyl compounds, which are significant aromatic components of the beverage (21). Alcohol oxidation, ketone acid decarboxylation, amino acid deamination, and decarboxylation metabolic pathways are some of the processes that can generate aldehydes and ketones (20). The fatty acid beta-oxidation process is the primary source of ketones, which give Baijiu its flowery, fruity, and creamy aromas. Ketones have a lower threshold value and a bigger contribution to aroma (22). Over 98% of the total aldehyde content in baijiu is made up of the primary aldehyde compounds, acetaldehyde and acetal. As storage time is extended, the amount of acetal in samples kept in Mare Nectaris increases; in older Mare Nectaris, the increase is more than in younger ones. Acetal is used to measure how mature baijiu is. New and old Mare Nectaris have variable amounts and ratios of acetaldehyde and acetal. Under various settings, Mare Nectaris can promote maturation, and new Mare Nectaris have a higher impact on carbonyl compounds in the baijiu, as evidenced by the mass concentration ratio of acetaldehyde to acetal in new baijiu, which is 1.19, 1.02 in NL6, 0.76 in NL12, 1.18 in OL6, and 1.06 in OL12. The levels of propionaldehyde and benzaldehyde (almond scent) are higher than in new baijiu, which is in line with other study findings. During the final stages of storage in new Mare Nectaris, isobutyraldehyde stays constant while progressively rising in old Mare Nectaris.

#### 3.2.4 Changes in major acid-derived flavor compounds

In Baijiu, organic acids are crucial flavoring ingredients. The baijiu can be made with the right amount and leave a lasting aftertaste. Lactic acid content rises and butyric acid content falls during storage in both new and old Mare Nectaris. The baijiu kept in the old Mare Nectaris for a year has a comparatively high lactic acid level. The fermentation and metabolism of lactic acid bacteria produce lactic acid, a non-volatile acid that gives Feng-flavor Baijiu its deep flavor. Lactic acid's resistance to volatility may be the cause of this. Furthermore, it has to do with the coating materials that are applied to the inner walls of the Mare Nectaris, like rapeseed oil, beeswax, and blood, all of which include some lactic acid (23, 24). The fermentation of ethanol produces acetic acid, which has a strong sour flavor. It has a comparatively high threshold and makes a negligible impact to baijiu's flavor. Over time, there is a more noticeable variation in the amount of acetic acid in the baijiu kept in both new and old Mare Nectaris. This could be because calcium salts of organic acids with more than three carbon atoms are insoluble, whereas only calcium acetate is soluble among the entire acid composition. Consequently, temperature fluctuations and the permeability of the Mare Nectaris have a stronger correlation with the volatility of acetic acid. The volatility of acetic acid varies with temperature and the holes in the coating on the inner walls of the Mare Nectaris. Baijiu's aging is a complicated process. The physical and chemical processes of esters, alcohols, and acids in Feng-flavor Baijiu can be accelerated by the migration of metal ions from the raw materials and natural esterification catalysts into the baijiu while it is stored in the Mare Nectaris. The aging mechanism and volatile components of Mare Nectaris are affected differently by different situations.

### 3.3 Volatile compound aroma activity values (OAVs)

Baijiu's aroma is most likely the product of a very intricate network of many taste components rather than the contribution of a single flavor molecule. In order to ascertain the significance and contribution of important volatile flavor chemicals in Feng-flavor Baijiu that was preserved in Mare Nectaris, OAV was computed (25). OAV ≥1 was deemed significant for flavor formation in Baijiu, and the OAV was used to evaluate the contribution of volatile chemicals to Feng-flavor Baijiu. Additionally, 31 main flavoring chemicals were found (Table 2).

**Table 2:** Aroma activity values (OAVs) of 31 volatile compounds in Feng-flavor Baijiu samples.

**Compounds**	**Notation^a^**	**Odordescription^b^**	**L0 OAV**	**NL6 OAV**	**NL12 OAV**	**OL6 OAV**	**OL12 OAV**
2,3-Butanediol	C1	Sweet	141.84	169.26	172.43	176.71	182.87
1,2-Propylene glycol	C2	Sweet	3.92	3.84	2.74	2.73	3.88
sec-butanol	C3	Fruity	3.11	3.32	3.14	3.33	3.17
n-Propanol	C4	Fruity, floral, grass	9.19	9.51	9.56	9.41	9.83
n-hexanol	C5	Floral	5.47	6.12	6.18	6.11	6.42
Isobutyl alcohol	C6	Malt	1.93	1.93	1.94	1.94	1.97
N-butyl alcohol	C7	Fruity	164.90	175.13	174.85	174.23	180.78
Active amyl alcohol	C8	Alcohol, fruity	2.14	2.24	2.23	2.29	2.35
Isoamyl alcohol	C9	Fruity, almond	1.37	1.47	1.46	1.46	1.50
Ethyl caproate	C10	Sweet, fruity, cellar, green melon	10,500.55	11,271.90	14,725.76	11,159.48	14,786.97
Ethyl heptanoate	C11	Floral, fruity, honey, sweet	1.19	1.33	1.68	1.25	1.51
Ethyl lactate	C12	Sweet, fruity, grass	13.24	15.07	15.78	15.88	19.09
Butyl caproate	C13	Pineapple	< 1	8.16	11.20	7.40	2.66
Ethyl caprylate	C14	Pear, lychee, sweet	1,375.38	1,515.82	1,911.37	1,409.97	2,005.15
Isoamyl caproate	C15	Apple, banana, pineapple	< 1	1.14	< 1	< 1	< 1
Isoamyl acetate	C16	Banana, sweet, apple	< 1	92.55	152.10	148.31	65.44
Ethyl valerate	C17	Peach, floral, sweet	1,388.43	1,426.31	1,782.70	1,446.67	1,758.64
Ethyl butyrate	C18	Apple, pineapple, floral	1,155.44	1,191.84	1,471.37	1,196.04	1,502.47
Ethyl palmitate	C19	Cream	< 1	8.88	8.89	8.79	9.20
Ethyl formate	C20	Aromatic	< 1	< 1	< 1	< 1	1.05
Ethyl acetate	C21	Pineapple, apple	40.14	36.73	41.82	38.63	46.04
Ethyl decanoate	C22	Pineapple, floral	< 1	6.89	1.14	< 1	1.72
Ethyl phenylacetate	C23	Rose, honey	< 1	< 1	1.89	< 1	5.86
Acetaldehyde	C24	Fruity	173.99	172.53	150.10	184.89	206.94
Propionaldehyde	C25	Grass	< 1	< 1	< 1	102.79	164.13
Acetal	C26	Fruity	9.12	10.54	12.41	9.80	12.18
Isovaleric aldehyde	C27	Floral, fruity	1,677.34	2,506.30	2,000.05	1,248.09	2,103.11
3-hydroxy-2-butanone	C28	Cream	11.48	5.33	< 1	10.96	8.54
Acetic acid	C29	Vinegar	3.33	3.57	3.58	3.66	3.50
Butyric acid	C30	Sweat, smell, pit mud	134.25	128.40	127.64	157.62	129.77
Hexanoic acid	C31	Sweat, animals, smell	93.17	98.51	94.50	104.69	94.47

Name: L0, base Baijiu; NL, new Mare Nectaris; OL, old Mare Nectaris.

OAV was calculated as the ratio of the mean of three replicates to the corresponding threshold based on data in the literature.

^a^C1–C31: odorant compounds in the sample.

^b^Odor description of the compound.

Alcohols: C1-C9; Esters: C10-C23; Carbonyls: C24-C28; Acids: C29-C31.

**Figure 11:**
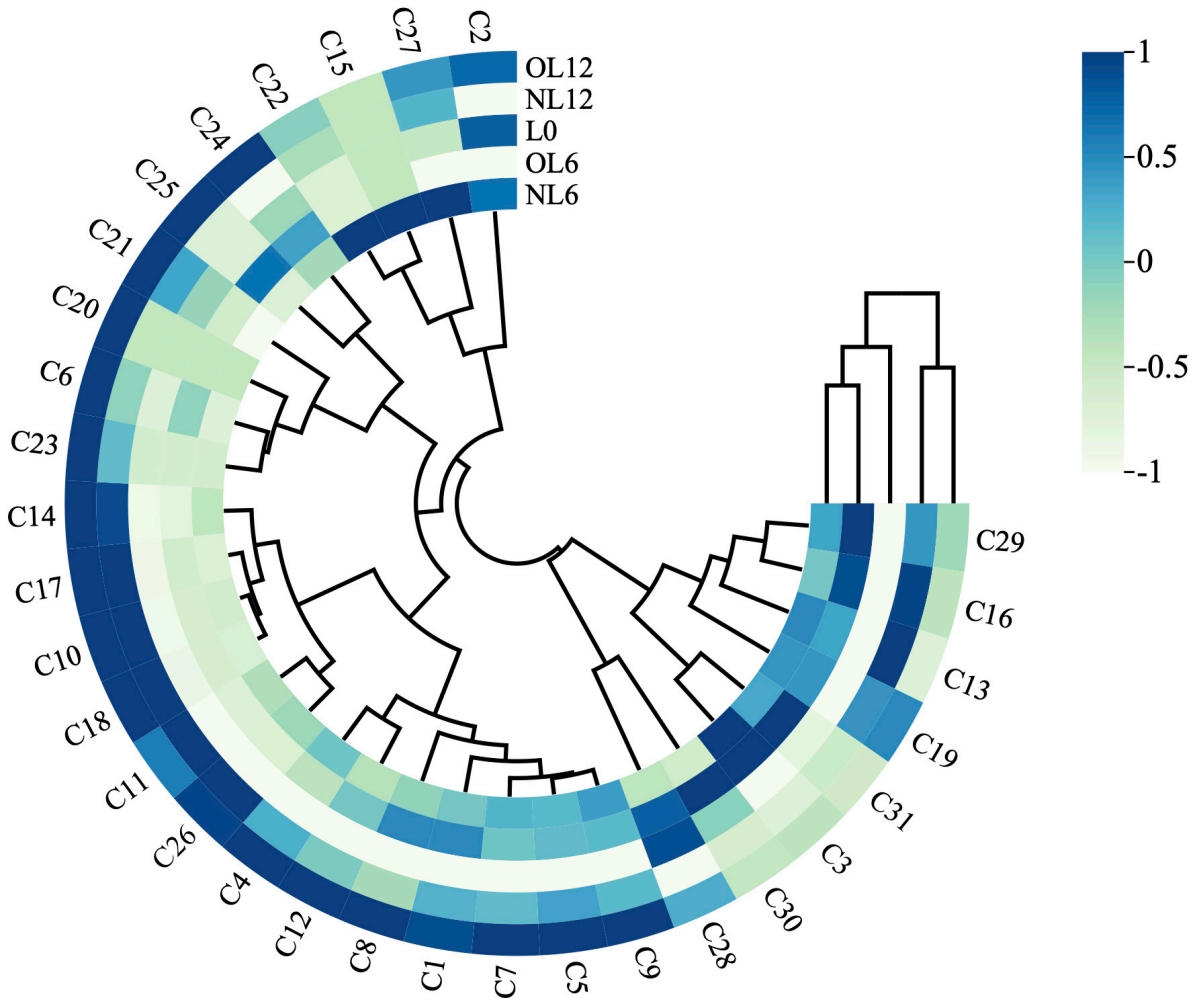
OAV heatmap analysis of Baijiu stored in different Mare Nectaris. Samples stored in new and old Mare Nectaris were designated “NL” and “OL”. The number after the letter indicates the storage time in months.

Table 2 displays each compound's OAVs. Nine alcohols, seven esters, three carbonyls, and three acids were among the twenty-three compounds with OAVs larger than one among the five Baijiu. Furthermore, as Figure 11 illustrates, NL6 had 28 compounds with OAVs >1, whereas OL12 had 30 compounds with OAVs >1. In accordance with the first classification results of flavor material content, Figure 11 illustrates the categorization of OAV in Feng-flavor Baijiu under various storage conditions: NL6 and OL6 are placed into one category, while NL12 and OL12 are placed into another.

The OAV of butyl caproate (C13), isoamyl acetate (C16), and ethyl palmitate (C19) in OL6, OL12, NL6, and NL12 were >1, indicating that the storage in the Mare Nectaris brought fruity and creamy aromas to the baijiu. Both OL12 and NL12 had OAVs of ethyl phenylacetate (C23) >1, with OL12 having a larger OAV than NL12, suggesting that the ancient Mare Nectaris could encourage honey discharge. The OAV of malondialdehyde (C25) in OL6 and OL12 was >100, and the malondialdehyde had a grassy aroma, which might be the key reason for OL12 to have a better Mare Nectaris aroma. The findings offer a theoretical foundation for improving the baijiu's storage procedure by showing that the length of storage and the degree of Mare Nectaris aging have a notable synergistic impact on the baijiu's flavor profile.

### 3.4 Correlation analysis of sensory and flavor substances in Feng-flavor Baijiu

**Figure 12:**
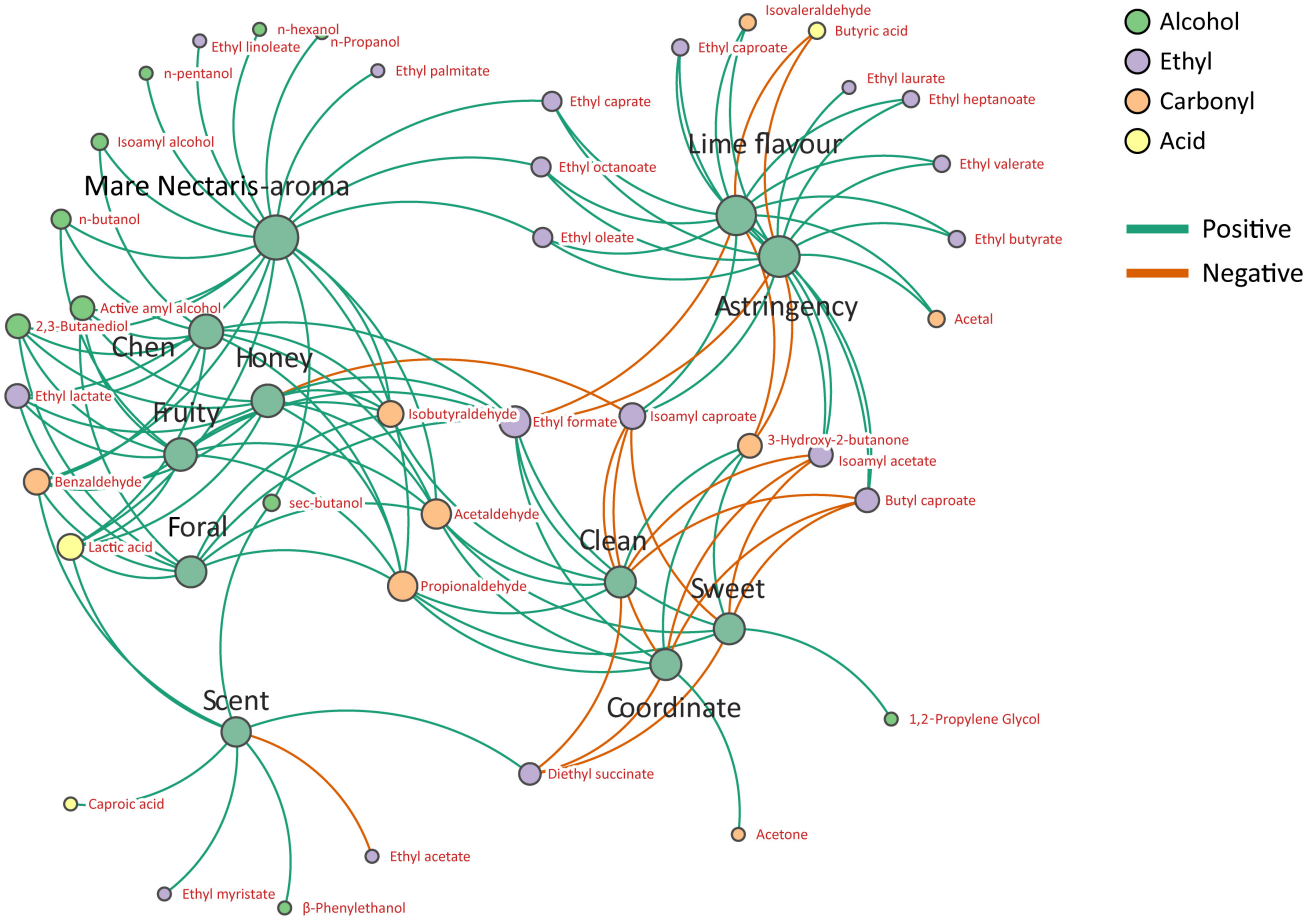
Network diagram of Spearman's correlation analysis between sensory and flavor substance of Feng-flavor Baijiu, the green and orange edges represent significant positive and negative correlations, respectively [Spearman's correlation coefficient (|*r*| > 0.7, *p* < 0.05)].

As shown in Figure 12, based on Spearman's correlation coefficient (|*r*| > 0.7, *p* < 0.05), the correlation analysis of sensory and flavor substances in Feng-flavor Baijiu with different storage conditions showed that the distance between the negative sensory attributes of lime taste and astringent taste was similar, the distance between sweet, clean and coordinate feeling was similar, and the distance between Mare Nectaris aroma, honey aroma, fruit aroma, flower aroma and old aroma was close. The positive correlation between sensory and flavor accounted for 84%, and the negative correlation accounted for 16%.

2,3-Butanediol, active pentanol, ethyl lactate, ethyl formate, acetaldehyde, propionaldehyde, isobutyraldehyde, benzaldehyde, lactic acid, n-butanol, and isoamyl alcohol had positive effects on honey, fruity, floral and Chen aroma, and isoamyl caproate was negatively correlated with honey. Among them, the active pentanol, acetaldehyde, propionaldehyde, lactic acid, ethyl formate, 2,3-butanediol, and benzaldehyde have a very significant effect on honey, floral, fruity, and Chen aroma.

2,3-Butanediol, sec-butanol, n-propanol, n-hexanol, n-butanol, active pentanol, isoamyl alcohol, n-amyl alcohol, ethyl lactate, ethyl caprylate, ethyl palmitate, ethyl oleate, ethyl linoleate, ethyl caprate, acetaldehyde, propionaldehyde, isobutyraldehyde, benzaldehyde, lactic acid were positively correlated with the aroma of Mare Nectaris, among which 2,3-butanediol, n-hexanol, n-butanol, active pentanol, isoamyl alcohol, ethyl lactate, ethyl palmitate, ethyl oleate, ethyl linoleate, acetaldehyde, propionaldehyde, isobutyl aldehyde, and lactic acid had a greater impact on the aroma of Mare Nectaris.

Ethyl hexanoate, ethyl heptanoate, butyl caproate, ethyl caprylate, isoamyl hexanoate, isoamyl acetate, ethyl valerate, ethyl butyrate, ethyl oleate, ethyl caprate, ethyl laurate, acetal, and c had a positive effect on the lime taste and astringency of the Baijiu, while ethyl formate, 3-hydroxy-2 butanone and butyric acid were negatively correlated with lime taste and astringency. Ethyl caproate, ethyl heptanoate, butyl caproate, ethyl caprylate, isoamyl caproate, and acetal were positively correlated with lime flavor and astringency.

Secondary butanol, ethyl tetradecanoate, diethyl succinate, benzaldehyde, and caproic acid were positively correlated with bloody odor. Butyl caproate, isoamyl caproate, isoamyl acetate, and diethyl succinate were negatively correlated with the sense of harmony and cleanliness of the bloody odor, indicating that the flavor substances were significantly affected by the lime and blood on the inner wall of the Mare Nectaris.

## 4 Conclusions

The dynamic transformation of Feng-flavor Baijiu's flavor components and sensory attributes over the course of Mare Nectaris' storage was methodically demonstrated by this investigation. The new Mare Nectaris gave the baijiu negative sensory qualities including “lime” and “astringent,” which made up its distinct fragrance identifier, according to sensory quantitative description analysis (QDA). Pleasant qualities like “honey,” “fruity,” and “Mare Nectaris-aroma” are greatly enhanced by the ancient astringent astringency, which helps to change the flavor of baijiu from jerky and stimulant to mellow and well-balanced. At the taste substance level, 45 critical aroma components were precisely measured using GC-FID and IC technology. The findings demonstrated that the old Mare Nectaris' storage conditions were more favorable for the buildup of fragrance compounds such ethyl lactate. The benefits of the old Mare Nectaris were further supported by the threshold-based Aroma Active Value (OAV) analysis, which showed that after a year of storage in the old Mare Nectaris, 30 compounds significantly affected the baijiu's overall aroma, compared to 27 compounds in the new Mare Nectaris. At the same time, it was discovered that the main indicators of flavor alteration were propionaldehyde, isoamyl acetate, isovaleraldehyde, and ethyl caproate. According to correlation studies, the properties of “honey” were primarily associated with lactic acid, active amyl alcohol, acetaldehyde, and propionaldehyde. In contrast, “Mare Nectaris-aroma” is more impacted by substances like propionaldehyde, ethyl lactate, ethyl palmitate, ethyl oleate, and ethyl linoleate. In conclusion, the aged Mare Nectaris has benefits in encouraging the buildup of flavor compounds and enhancing sensory quality, which offers a theoretical foundation for the control of aging and the enhancement of Feng-flavor Baijiu's quality.

## Data Availability

The original contributions presented in the study are included in the article/supplementary material, further inquiries can be directed to the corresponding author.
